# Metabolic Profiles Associated With Metformin Efficacy in Cancer

**DOI:** 10.3389/fendo.2018.00372

**Published:** 2018-08-21

**Authors:** Sylvia Andrzejewski, Peter M. Siegel, Julie St-Pierre

**Affiliations:** ^1^Department of Biochemistry, McGill University, Montreal, QC, Canada; ^2^Goodman Cancer Research Centre, McGill University, Montreal, QC, Canada; ^3^Department of Biochemistry, Microbiology and Immunology, and Ottawa Institute of Systems Biology, University of Ottawa, Ottawa, ON, Canada

**Keywords:** metformin, phenformin, mitochondria, diabetes, cancer, breast cancer, metabolism, mitochondrial drugs

## Abstract

Metformin is one of the most commonly prescribed medications for the treatment of type 2 diabetes. Numerous reports have suggested potential anti-cancerous and cancer preventive properties of metformin, although these findings vary depending on the intrinsic properties of the tumor, as well as the systemic physiology of patients. These intriguing studies have led to a renewed interest in metformin use in the oncology setting, and fueled research to unveil its elusive mode of action. It is now appreciated that metformin inhibits complex I of the electron transport chain in mitochondria, causing bioenergetic stress in cancer cells, and rendering them dependent on glycolysis for ATP production. Understanding the mode of action of metformin and the consequences of its use on cancer cell bioenergetics permits the identification of cancer types most susceptible to metformin action. Such knowledge may also shed light on the varying results to metformin usage that have been observed in clinical trials. In this review, we discuss metabolic profiles of cancer cells that are associated with metformin sensitivity, and rationalize combinatorial treatment options. We use the concept of bioenergetic flexibility, which has recently emerged in the field of cancer cell metabolism, to further understand metabolic rearrangements that occur upon metformin treatment. Finally, we advance the notion that metabolic fitness of cancer cells increases during progression to metastatic disease and the emergence of therapeutic resistance. As a result, sophisticated combinatorial approaches that prevent metabolic compensatory mechanisms will be required to effectively manage metastatic disease.

## Metformin

### A drug with a long history

Metformin was first discovered in the 1920s by a French physician from a plant called Goat's Rue (1). It was found that animals grazing on this plant had low blood glucose levels [reviewed in Witters (2)]. Subsequently, it was determined that the active compound responsible for lowering blood glucose was a guanidine moiety. Early synthetic homologues of guanidine were created for the treatment of diabetes, although they proved to be hepatotoxic and were rapidly discontinued. Renewed interest in guanidine in the 1960s led to the creation of a family of biguanide compounds [reviewed in White (3)]. Phenformin was the first biguanide family member prescribed to diabetic patients (4); however, its use was associated with the development of lactic acidosis (5). The biguanide metformin was better tolerated relative to phenformin by diabetic patients and was approved by the Food and Drug Administration (FDA) in the 1990s for the treatment of type 2 diabetes (6). Metformin is an extremely safe medication; rarely associated with the development of lactic acidosis (7). Additionally, metformin has global appeal as it is a low cost medication with generic versions also available.

It has been reported that patients with diabetes are more likely to develop cancer in their lifetime compared to non-diabetic individuals (8). A retrospective report published in 2005 suggested that metformin users have lower incidences of cancer relative to patients prescribed other type 2 diabetic medications (9). Moreover, users of diabetic medications other than metformin displayed increased cancer-related mortality (10). The study by Evans et al. (9) sparked great interest in the academic community, and metformin has been, or currently is being investigated in 310 individual clinical trials for its role in the prevention or treatment of various types of cancer. However, there is currently no consensus regarding which cancers are most likely to benefit from metformin treatment. Completed clinical trials have varied in outcome depending on trial design, cancer type, stage of cancer, timing of metformin treatment, and combinatorial therapies or treatments given in addition to metformin. Individual clinical studies have shown that metformin is associated with increased survival of diabetic patients with lung (11), colorectal (12), and prostate (13, 14) cancers. Moreover, metformin is associated with reduced risk of developing pancreatic (15), breast (16), colorectal (17) or liver (18) cancers. Recently, studies have been developed to investigate potential anti-cancer roles of metformin in non-diabetic patients given the increasing literature supporting its action in cancers, as well as the fact that metformin is associated with less hypoglycemic episodes than other diabetic medications (19). One randomized control trial on metformin monotherapy in advanced melanoma showed no benefit; however, the authors propose a more effective strategy would involve combining metformin with BRAF inhibitors and screening for patients with p53 polymorphisms (20). Such a trial in advanced melanoma has been completed (NCT02143050)^1^, and another combining metformin with cancer immunotherapy is ongoing (NCT03311308)^2^. One randomized trial of metformin combinatorial treatment with standard of care chemotherapy showed no benefit in advanced pancreatic cancer (21), despite large meta-analysis showing significant survival in metformin treated pancreatic patients (22). These studies highlight a need for more rigorous planning of clinical trials that focus more on potential predictive biomarkers (23). Additionally, a randomized trial with metformin monotherapy in early stage breast cancer is ongoing (NCT01101438)^3^, as well as a trial combining metformin with neo-adjuvant chemotherapy in HER2+ breast cancer (NCT03238495)^4^. These studies will reveal whether metformin's mode of action in cancer extends beyond its ability to reduce blood glucose levels, as glucose levels in healthy patients will not be affected by metformin treatment. Overall, the current available data support continued efforts toward examining the potential therapeutic role of metformin in various cancers, both in diabetic and non-diabetic patients.

### Molecular targets of metformin

**Figure 1:**
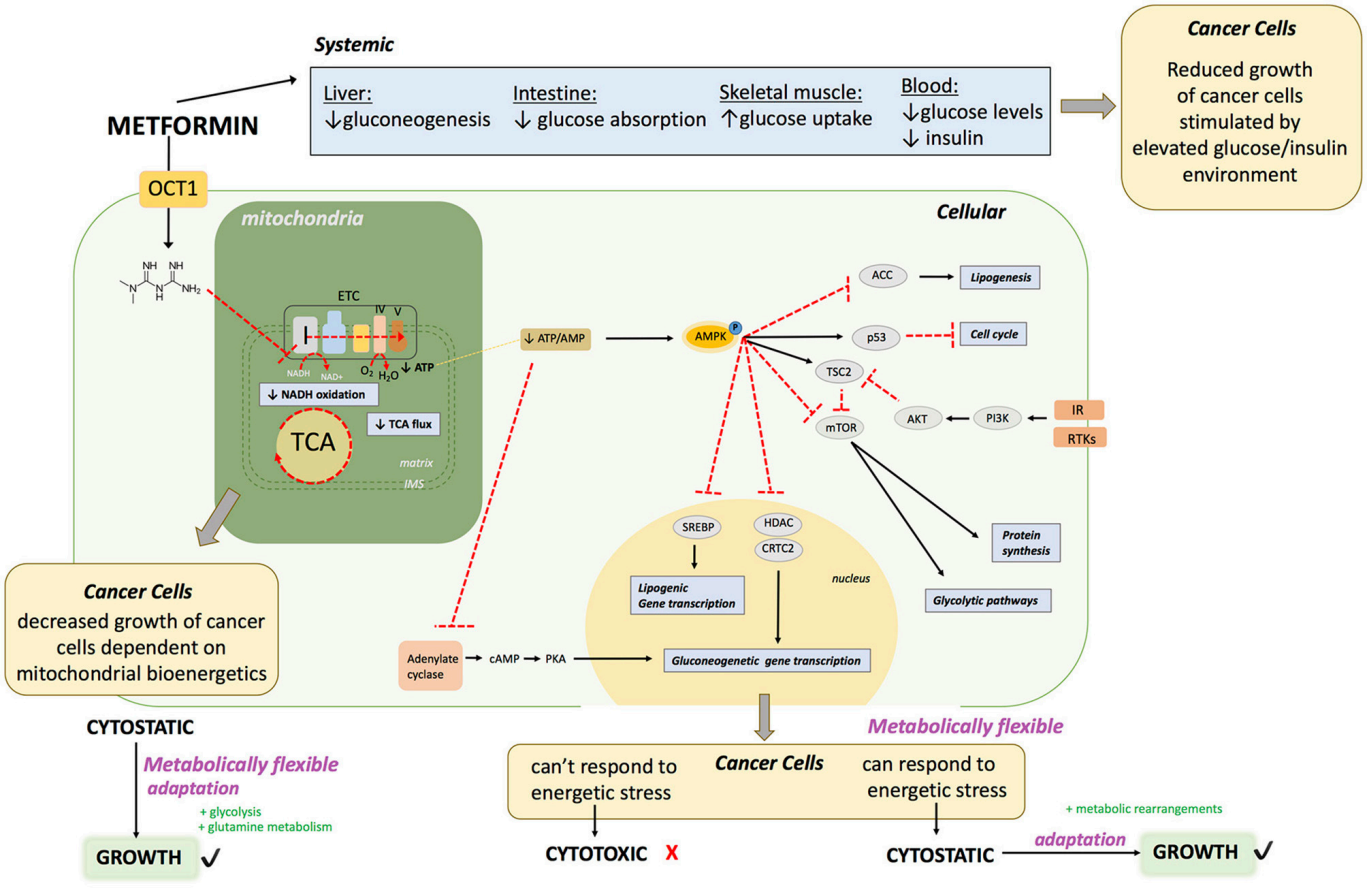
Proposed Molecular Mechanisms of Metformin Action.

Metformin is known to act on the liver, gut and skeletal muscle to globally lower blood glucose levels in diabetic patients with hyperglycemia (24) (Figure 1). The first report of a direct molecular target of metformin was in 2000 (25) showing that metformin acts on complex I of the electron transport chain (ETC) of mitochondria. However, the experiments in this study were performed under harsh experimental conditions that included incubation of mitochondria at low temperature (8°C) for extended periods of time (400 min) in the presence of high dose (10 mM) of metformin. The conclusions were rapidly challenged when a study showed that metformin had no direct effect on mitochondrial complex I (26). As a result, this controversy remained, and for over a decade following these initial observations the molecular mechanism of metformin was characterized as unknown or incompletely described. Various targets have been proposed by several groups, including complex II and IV of the ETC (27), LKB1/AMPK (28–30), adenylate cyclase (31), AMP deaminase (32), NADPH oxidase (33) and mitochondrial glycerophosphate dehydrogenase (34). Elucidation of a key molecular target of metformin came in 2014 when three groups, using differential approaches and experimental conditions published novel and conclusive evidence on the inhibitory properties of metformin on complex I (35–37). This included work on permeabilized cells and cancer cells that do not express complex I (37), isolated mitochondria (35, 36) and purified complex I (36). It is now generally accepted that a direct molecular target of metformin is complex I (24, 38, 39). Many of the other proposed effects and targets of metformin may be explained by a shift in NAD/NADH caused by complex I inhibition, leading to decreased activity of enzymes that depend on the fine balance of cellular NAD/NADH. Inhibition of mitochondrial glycerophosphate dehydrogenase could also perturb NAD/NADH (40). The controversies surrounding the action of metformin on cells may be partly explained by the varying concentrations used in experimental systems (28).

### Bioenergetic stress: metabolic disruption

Complex I is the entry point for reduced NADH in the ETC. Direct inhibition of complex I by metformin in cells decreases the proton gradient and mitochondrial oxygen consumption rate (35), diminishes tricarboxylic acid cycle (TCA) activity and metabolites (35, 41–45) and leads to decreased cellular ATP levels (25, 26, 43) (Figure 1). The inhibition of mitochondrial respiration and ATP production by metformin results in a compensatory increase in glycolysis (35, 43) as well as increased activity of glycolytic enzymes. These metabolic adaptations are engaged in an attempt to restore cellular ATP levels. However, if the compensatory activation of glycolysis cannot meet the cellular ATP requirements, AMPK becomes activated in order to potentiate catabolic metabolism, and inhibit anabolic reactions (30, 43, 47, 46). Phosphorylation and activation of AMPK leads to phosphorylation and inactivation of ACC, one of the most characterized targets of AMPK, causing a reduction in lipogenesis (48, 49). Metformin increases the levels of AMP causing inhibition of adenylate cyclase (31). Metformin also inhibits mTORC1 signaling (50, 51). Overall, metformin treatment causes at least a transient decrease in cellular energy status, leading to a global decrease in ATP consuming processes. In proliferating cells, this can elicit a cytostatic state that is associated with reduced proliferation, explaining some clinical observations of decreased progression of cancer cell growth. Cancer cells that cannot eventually compensate for this reduced energy status may undergo apoptosis (52, 53).

Metformin enters the cell via an OCT transporter; commonly OCT1 expressed on the surface of hepatocytes. Metformin acts directly on mitochondria to inhibit complex I of the ETC. This causes 1) diminished NADH oxidation at complex I, resulting in a buildup of NADH, 2) diminished TCA cycle activity due to allosteric inhibition of enzymes in the TCA cycle from increased NADH/NAD, 3) diminished flow of electrons throughout the ETC, and ultimately diminished oxygen consumption and ATP production at complex V (ATP synthase). This can lead to decreased growth of a subset of cancers that heavily rely on mitochondrial bioenergetics. Failure to rearrange metabolic programs leads to decreased ATP levels. Diminished ATP levels in the cell leads to AMPK activation. In hepatocytes, this drop in ATP leads to a decrease in gluconeogenesis due to allosteric inhibition of several leads to a decreased absorption of glucose. In the muscles, this leads to increased glucose uptake and eventually a decrease in hyperglycemia in the blood; with reduced glucose and insulin levels. The reduction in blood glucose and insulin levels may impair the growth of a subset of cancers that proliferate in an environment dictated by type 2 diabetes. At a cellular level, activation of AMPK leads (1) to inactivation of ACC, leading to a decrease in lipogenesis, (2) activation of p53 leading to a decrease in cell cycle progression, (3) inactivation of mTOR leading to decreased protein synthesis and glycolytic pathways. Inactivation of mTOR may be useful in a subset of cancers that have RTKs or IR activation. AMPK activation also leads to (4) decreased transcription of gluconeogenic genes by inhibition of HDAC and CRTC2, which is also achieved by (5) adenylate cyclase inhibition. Furthermore, AMPK activation causes (6) a decrease of lipogenic gene expression by inhibition of SREBP. The end result of metformin exposure is cellular energetic stress. If the cancer cells are metabolically flexible, allowing them to successfully respond to this stress by rearranging metabolic programs, metformin has a cytostatic effect, however if cells fail to cope, metformin has a cytotoxic effect.

**OCT1:** organic transporter 1, **TCA:** tricarboxylic acid cycle, **AMPK**: 5′ adenosine monophosphate- activated protein kinase, **IR:** insulin receptor **ACC** acetyl-CoA carboxylase: **mTOR:** mammalian target of rapamycin, **RTKs**: receptor tyrosine kinases. **cAMP:** Cyclic adenosine monophosphate, **AKT**: protein kinase B, **SREBP:** Sterol regulatory element binding protein, **HDAC:** histone deacetylase, **CRTC2:** CREB-regulated transcription coactivator 2.

## Metformin: bioenergetic medicine

### Bioenergetic medicine

Metformin is now classified as a bioenergetic disruptor and such drugs represent an exciting strategy to treat metabolic disorders, including cancer. Bioenergetic drugs affect ATP generating pathways, namely glycolysis, and oxidative phosphorylation. Bioenergetics is undeniably coupled to the proliferative potential of cancer cells. The focus of bioenergetic medicine (54) is not solely to impact ATP production, but also to disrupt biosynthetic pathways that rely on precursor metabolites found in ATP generating pathways for cancer cell proliferation. For example, glucose metabolism has been targeted using glycolysis inhibitors, such as 2-deoxyglucose (2-DG), a non-metabolizable glucose analog, which has been employed in clinical trials for various cancer types. Although many *in vitro* or murine studies demonstrate profound effects of 2-DG treatment on the growth of various cancer cell models (55–57), many clinical trials with 2-DG have been terminated early due to lack of early clinical efficacy as well as side effects, notably extreme exhaustion and cardiac arrhythmias in patients (NCT00633087)^5^. A completed study investigating an optimal dosage of 2-DG for solid tumors in combination with docetaxel treatment noted only moderate effects on stabilizing disease (58). However, significant side effects, including fatigue and nausea, were noted in many of patients (58).

In addition to glucose, many cancers are dependent on glutamine for their growth and are said to suffer from glutamine “addiction” (59). The expression of glutaminase is also up regulated in various cancer types (60–62). Murine tumor xenografts show promising anti-growth responses to inhibition of glutamine (glutaminase) metabolism (63, 64), and clinical trials are currently ongoing to test the efficacy of inhibiting glutaminase using a small molecule inhibitor (CB-839, Calithera Biosciences) in multiple types and stages of cancer (NCT02071862^6^; NCT02071888^7^; NCT03163667)^8^. It has also been suggested that metastatic progression is accompanied by increased glutamine utilization, and thus more aggressive prostate cancer cells were more sensitive to the glutaminase inhibitor CB-839 (65). However, to date, there are no glutaminase inhibitors approved for usage in cancer treatment.

### Sensitivity to metformin: a metabolic profile

**Figure 2:**
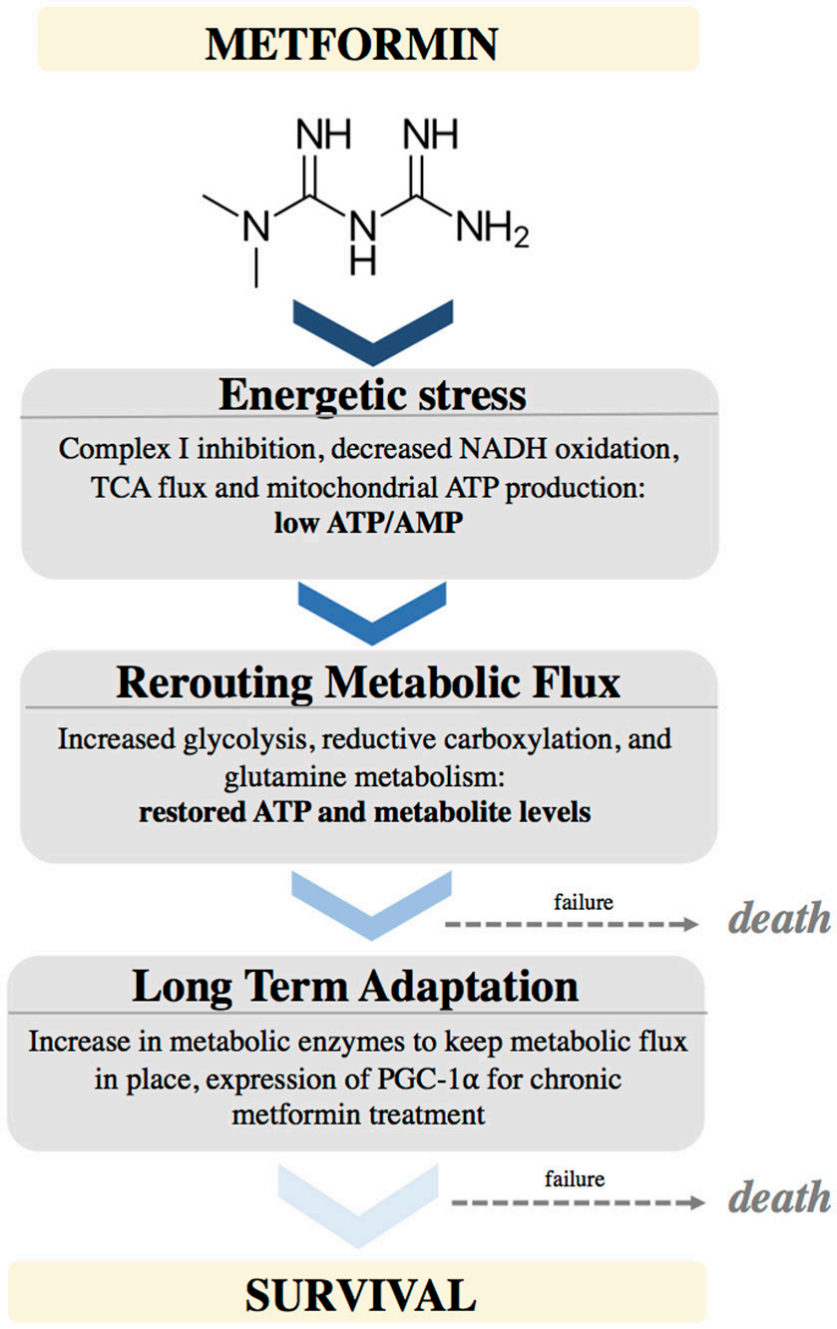
Schematic depicting the effects of Metformin on cellular metabolism and adaptations needed to support cancer cell survival.

Performing clinical trials in patients to determine which cancer type will benefit most from metformin treatment is undeniably important to understand the potential of this drug in oncology. With recent advancements, especially the identification of a molecular target of metformin, an alternative strategy to elucidate metformin's potential in oncology is to establish a “metformin sensitivity” profile at the cellular level to identify those cancer cell types most sensitive to its effects (Figure 2). This entails (1) understanding the metabolic changes that occur upon metformin treatment, (2) determining the cancer cell types most susceptible to these changes, (3) identifying those patients that would best benefit from metformin treatment and lastly, 4) defining combinatorial therapies that work best with metformin treatment in order to prevent compensatory mechanisms. This approach represents a rational and streamlined method to identify patients who would be most responsive to metformin treatment. However, it is difficult to predict whether the effects observed at the cellular level will translate *in vivo*. Therefore, the comparisons of the results obtained *in vitro, in vivo* and in clinical trials are necessary to reveal the full potential of metformin in the oncology setting.

To identify cancer cells most susceptible to metformin, we first need to recognize its mechanism of action and identify the internal cellular changes that occur upon treatment. Metformin inhibits complex I of the ETC in mitochondria, leading to perturbation in NAD/NADH and decreased oxygen consumption. This leads to diminished TCA activity and metabolite levels, as well as potential energetic stress leading to AMPK activation. Cells compensate for these metformin-mediated effects by increasing glucose uptake and glycolysis, and switching to glutamine utilization, as a way of refueling the TCA and providing biosynthetic intermediates for lipid production required to synthesize membranes (35, 42, 43, 66, 67). Hence, cancer cells exposed to metformin need to rearrange and reroute metabolic flux. It is increasingly evident that metformin alters substrate utilization in the mitochondria (45). As a result, cancer cells that would be most susceptible to metformin's action would have a high reliance on OXPHOS as a source of ATP and lack metabolic flexibility to efficiently engage glycolysis. For example, cancer cells with defective mitochondria may not be able to successfully switch mitochondrial substrate utilization due to mutations or defects in these metabolic pathways. As a result, cells with defective mitochondria could be more sensitive to metformin treatment due to their inability to alter mitochondrial substrate utilization. In support of this point, complex I mutations have been shown to predict sensitivity to phenformin (68). It is possible that cancer cells with oxidative phosphorylation deficits may thus be more sensitive to biguanides than normal tissues.

It is becoming more apparent that numerous metabolic programs and adaptations in cancer cells are mediated by the metabolic regulator PGC-1α (69). We predict that cancer cells expressing low PGC-1α levels, or that fail to upregulate PGC-1α in the presence of metformin, would be more sensitive as they may not efficiently engage adaptive programs to promote survival. Additionally, cancer cells with inactive or impaired AMPK signaling may be more sensitive to metformin treatment, as AMPK is the main energy sensor in the cell, and AMPK activation upon exposure to metformin contributes significantly to the upregulation of PGC-1α and its adaptive programs (70). Although previously controversial, AMPK is not required for metformin action; however, AMPK signaling is advantageous as an adaptive response to cope with energetic stress (71).

Metformin causes energetic stress in cells by inhibiting complex I of the electron transport chain in mitochondria. This causes a decrease in NADH oxidation, decreased TCA flux, leading to low levels of TCA metabolites. This causes a temporarily low ATP/AMP ratio. Cells react by rewiring metabolic flux. This includes up regulating pathways to support increased glycolysis, increased glutamine utilization to provide alternative sources of ATP as well as metabolites. Cells that fail to metabolically adapt to this stress will undergo cell death. After longer exposure to metformin, cells will adapt by stably increasing enzymes needed to maintain these metabolic pathways, partially by upregulating PGC-1α expression (Figure 2).

### Metabolic flexibility: targeting metformin resistance

It has recently been shown that chronic exposure to metformin in cancer cells ultimately leads to drug resistance and that this is linked to increased PGC-1α levels (41). Metformin resistant cells are metabolically flexible and able to switch fuel sources from oxidative metabolism to glycolysis and glutamine metabolism in the context of metformin-mediated inhibition of oxidative phosphorylation. Although at first it may seem counterintuitive to increase the level of PGC-1α, a key regulator of OXPHOS and mitochondrial biogenesis, upon inhibition of OXPHOS by metformin, it is now appreciated that PGC-1α clearly has functions outside of its classic role in mitochondrial metabolism. We argue that PGC-1α supports metabolic flexibility upon bioenergetic stresses. Elevated PGC-1α levels in the presence of metformin reprograms cellular metabolism and creates a new metabolic state that promotes an alternate source of ATP production through stimulation of glycolysis as well as facilitating anabolic metabolism by diverting mitochondrial metabolites that would normally be used for ATP production for use in anabolic reactions. In support of this point, PGC-1α controls numerous metabolic programs in cancer, notably glucose (41, 72), glutamine (73), fat (74), and one carbon metabolism (70). This ability of PGC-1α to support numerous metabolic programs in breast cancer cells allows for an enhanced fuel flexibility to cope with bioenergetic stressors such as metformin (41).

**Figure 3:**
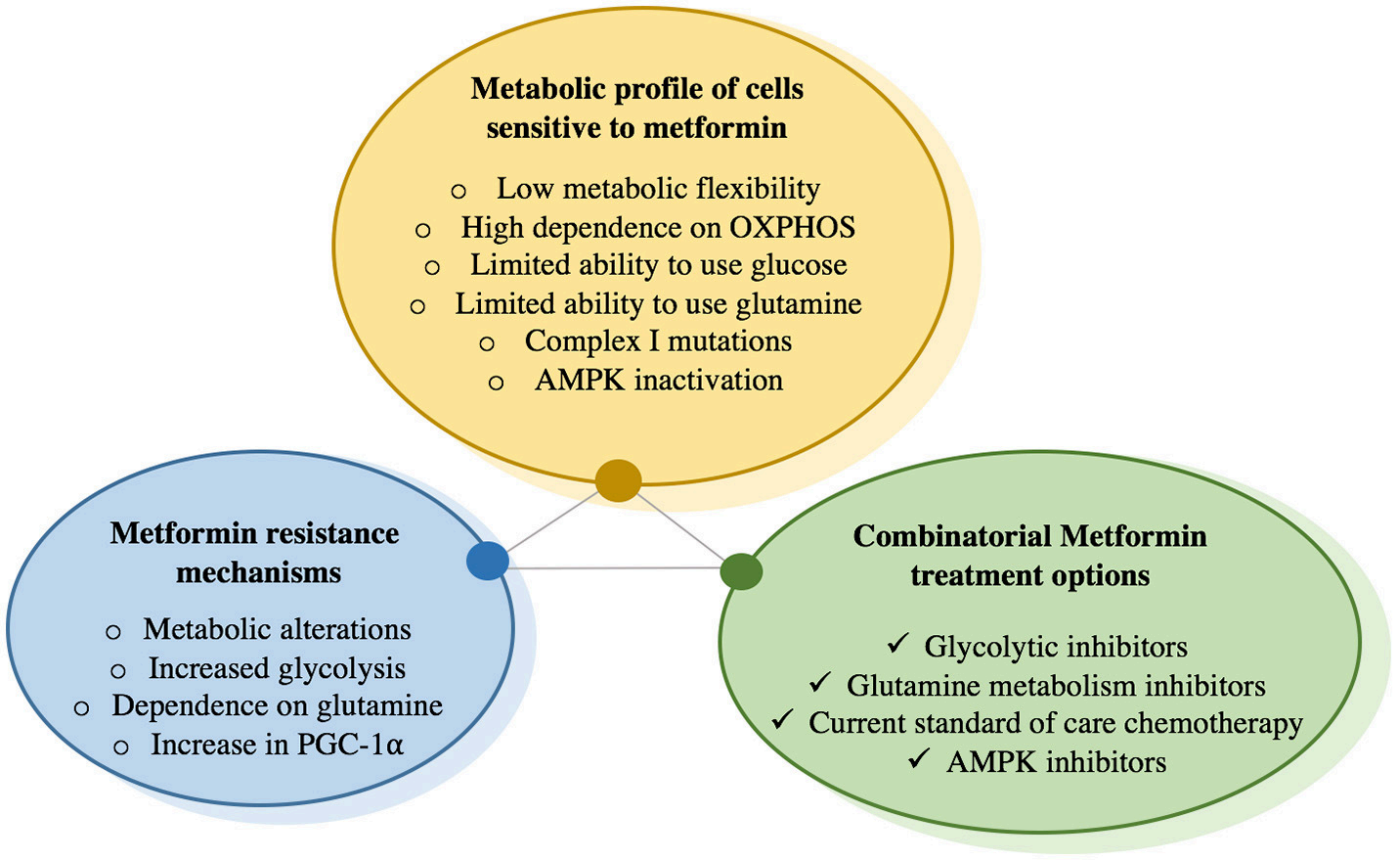
Concepts for metformin sensitivity, resistance, and combinatorial treatment options.

After developing a greater understanding of the metabolic rearrangements that occur upon metformin treatment (Figure 2), rational combinatorial treatments can be devised to combat adaptive mechanisms (Figure 3). The most immediate strategy would be to combine metformin with glycolysis inhibitors to prevent the adaptive glycolytic activity seen with metformin treatment alone. Blocking oxidative phosphorylation and glycolysis would stop the two main sources of ATP production, ultimately leading to cell death. Indeed, when breast cancer cells treated with metformin are deprived of glucose, this results in almost 100% cell death in just 72 h, even in the presence of glutamine (35). Additionally, it has been shown that cells with mutations leading to either impaired glucose utilization or mitochondrial DNA mutations are more sensitive to the effects of biguanides (68). Other reports have shown similar results by combining metformin with inhibitors of glycolysis and thus preventing ATP production (75, 76). One concern is that all cells are capable of engaging glycolysis and OXPHOS for ATP production, although their degree of dependence on either pathway can vary. Rapidly proliferating cells require much more ATP than differentiated cells, thus targeting ATP producing pathways could prove beneficial, as this rationale has been the basis of chemotherapy for decades. Another potential metabolic combination therapy could be the targeting of regulators of metabolic flexibility, notably PGC-1α. A small molecule compound was recently found to reduce PGC-1α-dependent gluconeogenic activity in the liver by increasing PGC-1α acetylation, leading to an amelioration of glucose homeostasis in a murine model of diabetes (77). However, the role of PGC-1α in gluconeogenesis is far more developed than in cancer, and cancer specific post-translational modifications on PGC-1α are far less understood.

In addition, metformin is currently used in clinical trials as a combinational therapy with already established treatment options, such as chemotherapy. It has been suggested that combinatorial treatment of metformin with chemotherapy may sensitize cancer cells to chemotherapy treatment, leading to improved treatment efficacy and lower doses of administered chemotherapy (78–82). In murine models, combinatorial metformin treatment with the chemotherapeutic agent doxorubicin led to reduced mammary tumor mass and relapse compared to either drug alone when performing xenograft experiments (83, 84). There is also data showing that metformin has synergistic effects with various chemotherapy agents, including Pemetrexed in cell lines of non-small cell lung cancer (NSCLC), (85) EGFR-TKI in patients with NSCLC (86), Trichostatin in osteosarcoma cell lines (87), Simvastatin in animal models of metastatic prostate cancer (88) and Nelfinavir in cervical cancer xenografts (89). It has also been suggested that metformin may lead to a re-sensitization of cancer cells that have become resistance to chemotherapy, the predominant cause of treatment failure in patients undergoing treatment (45, 84, 90, 91). One study showed that metformin reduces the differences in metabolism between chemotherapy resistant and sensitive cells (92). Furthermore, metformin was shown to target metabolic programs that chemoresistant cancer cells become reliant on, including OXPHOS and glutamine metabolism (92).

Overall, metabolic flexibility is required to adapt to bioenergetic stress, such as metformin exposure. Additionally, cancer cells treated with chemotherapeutic agents display vast metabolic arrangements enabling them to become resistant. Targeting this flexibility by inhibiting compensatory metabolic shifts, such as using inhibitors of glycolysis or glutamine metabolism, may prove useful. It is becoming clear that attacking only one aspect of cellular growth or one metabolic pathway will ultimately lead to metabolic rearrangements and the emergence of resistance. Targeting both cellular proliferation and metabolism could prove to be a more efficacious strategy. Another approach could be to overload the compensatory metabolic pathways by drastically increasing ATP demand through the use of chemotherapeutic agents.

The features of cancer cells that would make them most sensitive to metformin treatment are described. Cells become resistant to chronic exposure to metformin by increasing glucose uptake as well as glycolysis, increasing glutamine utilization as a vital metabolite precursor for biosynthetic needs, as well as increase in PGC-1α expression, which has been shown to increase metabolic flexibility that is needed to overcome metformin-mediated bioenergetic stress. To prevent compensatory mechanisms by cells exposed to metformin, this drug can be combined with glycolysis inhibitors that prevent metabolism of glucose to lactate, or glutamine metabolism inhibitors, which prevent glutamine utilization. There is also data suggesting that metformin has synergetic effects with certain chemotherapies and may re-sensitize cancer cells that have become resistant to chemotherapy.

## Future of metformin in oncology

### Development of novel complex I inhibitors in oncology

In addition to metformin, various mitochondrial drugs are being developed for potential uses in oncology, and have been shown to alter mitochondrial metabolism. These include: (1) small molecule BAY 87-2242 that was developed as a complex I inhibitor, leading to a reduction of melanoma tumor growth in murine models (93), (2) Xanthohumol that leads to the overproduction of ROS and eventual apoptosis in cancer cells (94), (3) Canagliflozin, a proposed inhibitor of complex I and mitochondrial glutamate dehydrogenase (95), which reduces the proliferation of prostate and lung cancer cells (96), 4] Fenofibrate, another proposed complex I inhibitor, that depletes cellular ATP and induces cytotoxicity in glioblastoma (97) and 5) small molecule inhibitor JC1-20679 developed to inhibit complex I, slowing the growth of a panel of cancer cell lines (98). These results highlight the importance of mitochondrial metabolism in cancer and support the notion of targeting mitochondria for cancer therapeutic purposes. At this stage, it is unknown whether some of these molecules will be approved for usage in clinical trials, as toxicity in humans has not yet been demonstrated for all these drugs. However, Canaglifozin is already used for the treatment of type 2 diabetes; but the FDA has recently added additional *Warning and Precautions* stating that this drug causes increased ketoacidosis, decreased bone density, and increased risk of leg and foot amputations (99, 100). Developing an effective drug for oncology is clearly not as simple as just synthesizing potent mitochondrial inhibitors. It is important to appreciate that complex I inhibitors, like rotenone or MPTP, can induce neurodegeneration in murine models (101, 102). However, metformin intake has been associated with better cognitive function in patients with Huntington's Disease (103). Indeed, it has been shown that metformin confers protection against mutant Huntingtin by modulating mitochondrial dynamics and activating AMPK (104).

In addition to metformin, phenformin is being revisited for usage in cancer therapy. Phenformin, like metformin, is a complex I inhibitor (36); however, it is transported with a greater affinity and kinetics into cells (105). For this reason, phenformin rapidly accumulates in cancer cells. Additionally, phenformin uptake will not depend on the genetic variation of transporters (OCT family), which have been shown to influence metformin uptake and efficacy due to individual polymorphisms (106). Phenformin is currently in a few clinical trials including a phase I clinical trial to determine optimal dosage for combined treatment with small molecule targeted therapies (Dabrafenib and Trametinib) for patients with BRAF mutated melanoma (NCT03026517)^9^. It is being examined whether phenformin can reduce melanoma resistance to traditional targeted therapies. It is possible that phenformin will become more rapidly used in future clinical trials; however, accurate dosage, which is effective yet minimizes side effects, has always been an issue, and is a key reason for its rapid discontinued use in diabetes (107). Therefore, there is still a need to determine optimal doses of phenformin for oncology application, while minimizing side effects such as lactic acidosis and gastrointestinal distress. With optimal dosage of phenformin, it may even be possible to decrease the dosage of chemotherapeutic agents.

Lastly, an emerging field in cancer metabolism is the development of organelle targeted therapeutics (108), which could be utilized to specifically localize and compartmentalize therapies to potentially minimize adverse effects. This notion could be used to reduce administered doses of therapy, while maximizing dose in the compartmentalized region, although this research area needs to be developed further.

## Author contributions

SA wrote most of the text for this manuscript. SA, PS and JS-P contributed to the concepts, writing and editing of the manuscript.

### Conflict of interest statement

The authors declare that the research was conducted in the absence of any commercial or financial relationships that could be construed as a potential conflict of interest.

## Footnotes

^1^NCT02143050 Study of Dabrafenib, Trametinib and Metformin for Melanoma Patients., (https://clinicaltrials.gov/show/NCT02143050).

^2^NCT03311308 A Trial of Pembrolizumab and Metformin Versus Pembrolizumab Alone in Advanced Melanoma., (https://clinicaltrials.gov/show/NCT03311308).

^3^NCT01101438 A Phase III Randomized Trial of Metformin vs Placebo in Early Stage Breast Cancer., (https://clinicaltrials.gov/show/NCT01101438).

^4^NCT03238495 Randomized Trial of Neo-adjuvant Chemotherapy With or Without Metformin for HER2 Positive Operable Breast Cancer., (https://clinicaltrials.gov/show/NCT03238495).

^5^NCT00633087 A Phase I/II Trial of 2-Deoxyglucose (2DG) for the Treatment of Advanced Cancer and Hormone Refractory Prostate Cancer. (https://clinicaltrials.gov/show/NCT00633087).

^6^NCT02071862 Study of the Glutaminase Inhibitor CB-839 in Solid Tumors.

^7^NCT02071888 Study of the Glutaminase Inhibitor CB-839 in Hematological Tumors.

^8^NCT03163667 CB-839 With Everolimus vs. Placebo With Everolimus in Patients With RCC.

^9^NCT03026517 Clinical Trial of Phenformin in Combination With Dabrafenib and Trametinib for Patients With BRAF-mutated Melanoma.
